# Isoforms in action: The impact of transcript diversity in phenotype

**DOI:** 10.1590/1678-4685-GMB-2025-0220

**Published:** 2026-05-15

**Authors:** Izabela Mamede, Carlos Mata-Machado, Clarisse Reis, Paulo P. Amaral, Gloria R. Franco

**Affiliations:** 1 Universidade Federal de Minas Gerais, Instituto de Ciências Biológicas, Departamento de Bioquímica e Imunologia, Laboratório de Genética Bioquímica, Belo Horizonte, MG, Brazil.; 2 IDOR - Instituto D’Or de Pesquisa e Ensino, São Paulo, SP, Brazil.; 3 INSPER - Instituto de Ensino e Pesquisa, São Paulo, SP, Brazil.

**Keywords:** RNA isoforms, splicing, regulatory RNA, noncoding RNA, lncRNA

## Abstract

The study of RNA processing, in particular how alternative splicing and the use of alternative transcription start and end sites generate diverse transcript isoforms from a single gene, has advanced significantly alongside the development of RNA sequencing technologies. High-resolution whole-transcriptome sequencing has shown that much of the transcriptomic diversity observed in humans, compared to other species, stems from these alternative RNA processing events. Primary studies first revealed that a single gene could give rise to multiple RNA variants, which challenges the linear interpretation of the central dogma of molecular biology. However, progress in the field was limited by technological constraints and the inherent complexity of resolving isoforms at high resolution. Recent breakthroughs, which includes long-read sequencing, high-throughput FISH-based imaging, isoform-specific quantification tools, and improved software for transcript detection and quantification, have reignited interest and enabled a more detailed view of transcriptomic diversity. Understanding alternative isoforms is significant, as they expand the proteome, fine-tune gene regulation, and influence cellular phenotypes that are key to human disease research and possible therapeutic development.

## Introduction

**Figure 1 -:**
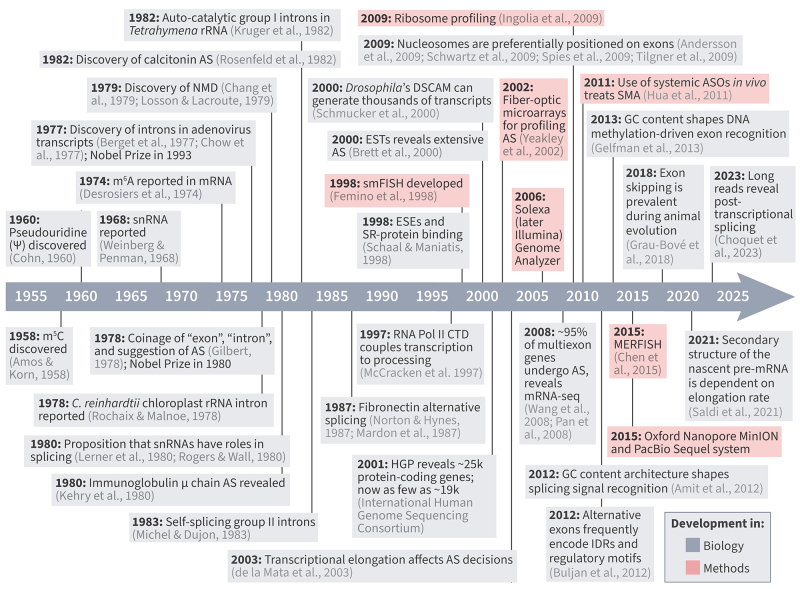
Historical milestones in RNA splicing and isoform biology. Timeline highlighting key discoveries and technological advances that shaped the field of RNA splicing. Early findings in the 1960s-1970s established the presence of RNA modifications (m^5^C, m^6^A, pseudouridine) and small nuclear RNAs (snRNAs), followed by the landmark discovery of introns in adenoviral transcripts in 1977, which overturned the linear view of the central dogma and introduced the concept of splicing. The 1980s and 1990s revealed biological functions of alternative splicing (AS), including immunoglobulin heavy chain and calcitonin/CGRP isoforms, and identified catalytic RNAs and nonsense-mediated decay (NMD). From the late 1990s onward, large-scale sequencing (ESTs, microarrays, Human Genome Project) and computational methods revealed the pervasiveness of AS across the human genome. In parallel, imaging approaches such as smFISH and MERFISH enabled visualization of isoforms in situ. The 2000s-2010s brought high-throughput methods including ribosome profiling and next-generation sequencing (Illumina, PacBio, Oxford Nanopore), which transformed transcriptome-wide isoform analysis. Subsequent studies established coupling of RNA polymerase II via its carboxy-terminal domain (CTD) to splicing, and showed that elongation rate and chromatin organization influence AS. The beginning of the following decade was distinguished by the use of antisense oligonucleotides (ASOs) to treat spinal muscular atrophy (SMA). Most recently, long-read sequencing and direct RNA profiling have uncovered widespread post-transcriptional splicing and demonstrated that retained introns (RI) can remain stable as part of RNP complexes. Developments in biology are shown in gray, while methodological advances are shown in pink (Ingolia et al., 2009; Grau-Bové et al., 2018; Saldi et al., 2021).

Before 1977, the prevailing view of the central dogma of molecular biology was linear: genes were transcribed into messenger RNAs (mRNAs), which were then translated into proteins, with little perceived complexity between transcription and translation. mRNAs were known to be chemically modified (Amos and Korn, 1958; Desrosiers et al., 1974; Cohn, 1960) and small RNAs of unknown function had been found in the nucleus (Weinberg and Penman, 1968) but these observations were generally interpreted as biochemical features of RNA. Then two independent groups (Phillip Sharp’s team at MIT (Berget et al., 1977) and Richard Roberts’s (Chow et al., 1977) group at Cold Spring Harbor) demonstrated that adenoviral mRNAs did not correspond to uninterrupted stretches of DNA. Instead, their transcripts were composed of coding segments (later termed exons) interspersed with noncoding sequences (introns) that were removed before translation (Gilbert, 1978). These findings earned Sharp and Roberts the 1993 Nobel Prize in Physiology or Medicine and established the concept of RNA splicing (Figure 1). In the years immediately following the discovery of introns, RNA processing was rapidly linked to phenotype. Studies showed that nonsense mutations could influence gene output through effects on RNA, from altered clinical expression in β-thalassaemia (Chang et al., 1979) to reduced mRNA stability in yeast, anticipating later concepts such as nonsense-mediated decay (Losson and Lacroute, 1979). Early models of the splicing machinery also emerged, proposing roles for those small nuclear RNAs (snRNPs) in the process (Lerner et al., 1980) and outlining mechanistic steps by which RNA splicing could occur (Rogers and Wall, 1980).

If introns could be selectively removed, then different combinations of exons could, in principle, be joined together, enabling the production of multiple mRNA isoforms from a single gene. On the other hand, biological processes are not perfect, so the production of RNAs could also keep these introns into mature non-translatable transcripts. This process, later termed alternative splicing (Breitbart et al., 1987), provided a mechanism for greatly expanding the coding potential of genomes without increasing the gene number. Early experimental evidence of alternative splicing soon followed. In the early 1980s, studies of the immunoglobulin heavy chain gene in B lymphocytes revealed that the same pre-mRNA could be spliced differently to generate either a secreted or membrane-bound antibody isoform, depending on the stage of B-cell differentiation (Early, 1980; Kehry et al., 1980). Around the same time, alternative splicing was also documented in calcitonin and calcitonin gene-related peptide (CGRP) transcripts, showing that tissue-specific splicing could generate peptides with entirely distinct biological functions (Rosenfeld et al., 1981, 1982). These discoveries shifted the perception of RNA processing from a passive mechanism to an active regulator of gene expression. Ribosomal RNA was also found to be self-spliced (Rochaix and Malnoe, 1978; Kruger et al., 1982) and some RNA secondary structures were conserved in the chloroplast, mitochondria and nucleus (Michel and Dujon, 1983). The concept that a single gene can give rise to multiple proteins (sometimes with divergent, even opposing roles) challenged the “one gene, one protein” paradigm and laid the foundation for isoform biology.

By the late 1990s and early 2000s, it became clear that alternative splicing was not an uncommon event, but rather a widespread mechanism shaping eukaryotic transcriptomes. Transcription and RNA processing were found to be coupled processes, with RNA pol II mutants affecting transcriptional elongation (Chen et al., 1996; de la Mata et al., 2003) and pol II carboxyl-terminal domain (CTD) directly interacting with 3’ splicing factors (McCracken et al., 1997). Expressed sequence tag (EST) projects, and high-throughput cDNA sequencing, revealed that a substantial fraction of human genes produced multiple isoforms (Brett et al., 2000). Early computational analyses suggested that ~40% of human genes underwent alternative splicing (Mironov et al., 1999; Modrek, 2001), a number that would later increase with the advent of RNA-seq. This discrepancy was in part associated with the exonization of Alu elements due to specific mutations (Lev-Maor et al., 2003), that are widespread in mammalian genomes.

The functional consequences of this diversity also became clearer. Alternative splicing was shown to influence virtually all aspects of gene expression, from protein domain architecture (by including or excluding coding exons) to mRNA stability (via nonsense-mediated decay) and protein cellular localization (Choi et al., 2023). Classic developmental examples were described, such as *Drosophila melanogaster Dscam* (Down Syndrome Cell Adhesion Molecule), gene, which can generate tens of thousands of isoforms through splicing of mutually exclusive exons, contributing to neuronal wiring specificity (Schmucker et al., 2000; Neves et al., 2004) or also *D. melanogaster* sex determination by alternative splicing of *Sxl* (Sex-lethal), *Tra* (Transformer) and *Dsx* (Doublesex) transcripts (Boggs et al., 1987; Bell et al., 1988; Ryner and Baker, 1991). In mammals, the Richard O. Hynes and Francesco Baralle groups described alternative exon usage in the large extracellular matrix glycoprotein fibronectin (FN1 gene). This became a classical example of alternative splicing, since different tissues and phases in cellular development keep different FN1 exons after RNA processing (Kornblihtt et al., 1984, 1985; Mardon et al., 1987, Norton and Hynes, 1987). 

The field also converged on the idea that transcriptional regulation and chromatin state shape splice-site choice. Studies of nuclear receptor signaling provided early evidence that differential recruitment of transcriptional coactivators influence splice-site selection (Auboeuf et al., 2004; Schaal and Maniatis, 1999). The chromatin remodeling factor SWI/SNF, subunit BRM was shown to regulate splicing outcomes via effects on elongation dynamics (Batsché et al., 2005). Multiple studies in 2009 also reported that nucleosomes are preferentially positioned on exons and that exons carry specific chromatin signatures (Andersson et al., 2009; Schwartz et al., 2009; Spies et al., 2009; Tilgner et al., 2009). Subsequent work distinguished when an alternative exon is “committed” to be included, arguing in favor of a “first committed” framework rather than a “first excised” model (de la Mata et al., 2010). In addition to altering domain architecture and mRNA fate, alternative splicing expands the functional repertoires of intrinsically disordered protein regions (IDRs) and regulatory motifs (Buljan et al., 2012). Early evidence indicated that alternative splicing in concert with IDRs can increase functional diversity in multicellular organisms (Romero et al., 2006), and later analyses supported that alternative exons frequently encode disordered segments and regulatory motifs (Amit et al., 2012). 

By the mid-2000s, the emerging consensus was that alternative splicing represented a central layer of gene regulation, shaping proteomic diversity far beyond what gene count alone could explain (Pan et al., 2008; Wang et al., 2008). These insights became especially important following the Human Genome Project’s revelation that humans possess only about 20,000 protein-coding genes-comparable to the nematode *Caenorhabditis elegans* (The *C. elegans* Sequencing Consortium, 1998). Today, that number is estimated to be closer to 19,000 (Amaral et al., 2023). The disparity between gene number and organismal complexity highlighted the central role of alternative splicing in enhancing regulatory and functional diversity. At the time, most transcriptome studies relied on short-read sequencing or microarray gene expression analysis (Yeakley et al., 2002). While these methods were powerful for detecting differential gene expression, they lacked the resolution to unambiguously reconstruct full-length isoforms. Reads of 50-150 base pairs could identify splice junctions but assembling them into complete isoforms across multi-exonic genes was computationally challenging and error prone. Part of this difficulty reflects that splice-site recognition operates under sequence architectures, including GC-content differences between exons and introns that bias recognition strategies (Amit et al., 2012). These constraints also interact with epigenetic regulation: for example, DNA methylation effects on cotranscriptional splicing have been reported to depend on the GC organization of the exon-intron structure (Gelfman et al., 2013).

As a result, most of the studies collapsed signals into gene-level expression, effectively ignoring the complexity of isoform usage. Bioinformatics also lagged behind the complexity of the problem. Early algorithms for transcript assembly (e.g., Cufflinks (Trapnell et al., 2010), Scripture (Guttman et al., 2010), rMATS (Shen et al., 2014)) and isoform quantification produced conflicting results and were sensitive to sequencing depth and annotation completeness. Without standardized pipelines or reliable benchmarks, isoform-level findings were often viewed with skepticism. This analytical bottleneck meant that, despite occasional reports of isoform switches in development or disease, the field lacked systematic catalogs of isoform diversity.

The literature during this period was dominated by isolated case studies of well-known genes, such as *BCL2L1* (BCL2-like-1), previously known as *BCL-X*), which produces pro- and anti-apoptotic isoforms (Aird et al., 2019); or *PKM* (Pyruvate Kinase M), where *PKM1*/*PKM2* splicing alters metabolic programs (Wang et al., 2012), rather than genome-wide inferences.

Another limitation was the difficulty in linking isoform diversity to functional outcomes. Even when alternative splicing was detected, it was not clear whether resulting isoforms were stable, translatable, or even biologically relevant. This uncertainty fueled debates about whether most splicing was functional or merely “noise” (Mattick, 2004). Therefore, while the importance of alternative splicing was widely acknowledged, the field saw little integrative progress for some time. It was only with the advent of new sequencing technologies and imaging approaches in the middle 2010s, these able to capture transcripts end-to-end and quantify them accurately, that isoform biology entered its current era.

The introduction of third-generation sequencing platforms transformed the ability to study isoforms. Unlike short-read technologies, Pacific Biosciences (PacBio) Single Molecule Real-Time (SMRT) sequencing (Roberts et al., 2013) and Oxford Nanopore Technologies (ONT) platforms generate reads that can span entire RNA molecules (Soneson et al., 2019). This capability enables unambiguous identification of exon connectivity, alternative transcription starts and end sites, and even post-transcriptional modifications. PacBio Iso-Seq protocol was among the first to provide high-quality, full-length transcriptomes (Gonzalez-Garay, 2016). Its circular consensus sequencing approach reduces error rates and allows robust detection of rare isoforms, making it widely adopted in reference transcriptome projects. ONT, in contrast, offers the unique possibility of direct RNA sequencing, where native RNA molecules are sequenced without prior reverse transcription. This preserves information about RNA modifications and poly(A) tail length, adding another layer to isoform biology (Chen et al., 2025 c ). Studies across human tissues and model organisms consistently show that the majority of multi-exonic genes express multiple isoforms (Mudge et al., 2025). Importantly, long-read sequencing has confirmed that many alternative isoforms are capable of being translated into functional proteins, generate stable noncoding RNAs, or regulatory transcript variants with distinct subcellular fates. In parallel, advances in imaging-based methods brought isoform biology into the context of tissues and single cells. Techniques such as single-molecule FISH (smFISH) (Femino et al., 1998) and its massively multiplexed derivatives (including MERFISH (Multiplexed Error-Robust FISH) (Chen *et al*., 2015) and seqFISH (Lubeck et al., 2014) allow direct visualization of transcripts at isoform resolution inside intact cells. These methods rely on designing probe sets that specifically target exon-exon junctions or isoform-unique sequences, enabling researchers to distinguish between splice variants *in situ*.

While long-read sequencing and imaging technologies made isoforms visible, equal progress came from computational methods that allowed their systematic quantification, statistical testing, and integration across datasets. The introduction of pseudoalignment-based quantifiers provided direct transcript quantification. Traditional short-read alignment required mapping each read to the genome, which was computationally expensive and ambiguous at exon junctions. Tools like Kallisto (2016) (Bray et al., 2016) and Salmon (2017) (Patro et al., 2017) introduced the concept of pseudoalignment to a transcriptome index. These methods not only reduced runtime and memory requirements but also enabled robust isoform-level quantification based on previous long-read annotated transcriptomes directly from short-read data. Salmon has since incorporated advanced features such as bias correction (GC content, fragment position) and bootstrapping for inferential replicates, which are critical for differential transcript usage (DTU) testing. With accurate quantification came the need for statistical models tailored to isoforms. Classical differential expression packages like edgeR (Robinson et al., 2010) and DESeq2 (Love et al., 2014) were developed for gene level. To extend them, new approaches emerged like Sleuth (Pimentel et al., 2017), fishpond (Zhu et al., 2019) and the newer versions of edgeR (Chen et al., 2025 b ). These advances made it possible not only to quantify isoforms, but also to test whether specific splicing events or isoform shifts are statistically significant across biological conditions.

From a disease perspective, the consequences are profound. Isoform dysregulation has been implicated in cancer (Bradley and Anczuków, 2023), neurodegeneration (Nikom and Zheng, 2023), and immune disorders (Ren et al., 2021). Soluble *FLT1* (Fms Related Receptor Tyrosine Kinase 1) isoforms contribute to preeclampsia (Rajakumar et al., 2009), *TP63* (Tumor Protein 63) isoforms can drive oncogenesis (Chen et al., 2025 a ), and *NEAT1* (Nuclear-Enriched Abundant Transcript 1) isoforms reshape nuclear architecture in response to cellular stress (Knutsen et al., 2022). A clinical example is spinal muscular atrophy (SMA), a disease usually caused by the biallelic loss-of-function of the *SMN1* (Survival Motor Neuron 1) gene which leads to a deletion of its exon 7. In the human genome there is a paralogue of *SNM1*, *SNM2* (Survival Motor Neuron 2). Splice-switching antisense oligonucleotides (ASOs) were developed to promote the inclusion of this exon 7 in the *SMN2* gene, which in turn restores the function of the SMN protein (Hua et al., 2008), Systemic dosing in a severe SMA mouse model produced phenotypic rescue, establishing *in vivo* feasibility for AS-mediated therapy (Hua *et al*., 2011). This led to the approval of the first splicing-modulating therapy for SMA (Rigo et al., 2012).

We and others (Mattick, 2023) argue that the primordial role of RNA is regulatory, functioning as a noncoding molecule, with protein-coding capacity emerging later. While a subset of transcripts indeed encodes proteins, most coding loci also generate noncoding isoforms, often in greater numbers than their protein-coding counterparts. This balance, or shift, between coding and noncoding isoform production represents a critical regulatory axis underlying many human phenotypes.

**Figure 2 -:**
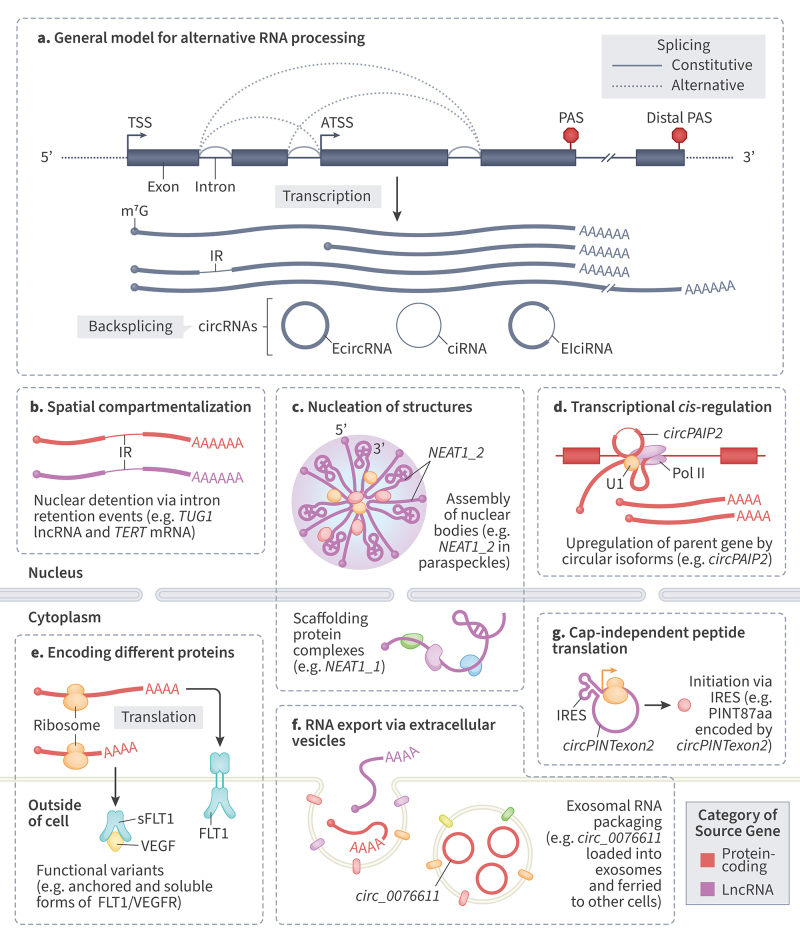
Functional outcomes of alternative RNA processing and isoform diversity. (a) General model of alternative RNA processing showing alternative transcription start sites (TSS/ATSS), splicing (constitutive vs. alternative), intron retention (IR), alternative polyadenylation sites (proximal/distal PAS), and backsplicing to generate circRNAs (EcircRNA, ciRNA, EIciRNA). (b) Spatial compartmentalization: intron-retained (IR) isoforms can be detained in the nucleus (e.g., *TUG1*, *TERT*). (c) Nucleation of nuclear bodies: long *NEAT1_2* scaffolds paraspeckles, recruiting DBHS proteins. (d) Transcriptional cis-regulation by circular isoforms: circRNAs (e.g., *circPAIP2*) interact with U1/Pol II to enhance parental-gene transcription. (e) Encoding different proteins: alternative 3′ end formation produces soluble vs membrane-anchored receptor isoforms (e.g., sFLT1 vs FLT1), modulating VEGF signaling. (f) RNA export via extracellular vesicles: full-length isoforms and circRNAs (e.g., *circ_0076611*) are packaged into exosomes for intercellular communication. (g) Cap-independent translation: Internal Ribosome Entry Site (IRES)- or m^6^A-mediated initiation in linear or circular RNAs yields peptides (e.g., *circPINTexon2* coding for PINT87aa). Color key indicates the category of the source gene of each isoform (protein-coding vs. lncRNA).

In this review, we synthesize how isoforms shape molecular phenotypes across both coding and noncoding transcripts, with emphasis on cases where isoform diversity directly influences disease mechanisms. Together, these studies demonstrate that alternative isoforms are not merely byproducts of transcriptional complexity, but rather essential regulators of cellular function and disease outcome (Figure 2).

## Alternative isoforms of coding genes

Alternative transcript isoforms of protein-coding genes dramatically expand the functional repertoire of proteins coded by the genome (Figure 2 A ). Recent advances in 3D protein predictions allowed a group led by Steven Salzberg to execute structural predictions using AlphaFold2 (Jumper et al., 2021) for all the annotated human protein coding isoforms (Sommer et al., 2022). Most of these isoforms are a product of alternative transcription start and end sites, as well as alternative splicing, where they result in protein domains being lost, but maintaining the reading frame. The most notable and explored example of this phenomenon happens in the Growth Factor Receptor family. Members of this family, when in the canonical protein form, possess three distinct domains: (1) an Immunoglobulin-like (Ig-like) domain that is located outside the cellular membrane, (2) a transmembrane domain that anchors the receptor to the cellular membrane and a (3) tyrosine kinase domain that activates the proliferation cascade when the ligand interacts with the Ig-like domain (Xie et al., 2020). On *FLT1*, previously known as *VEGFR*, there is an alternative poly-adenylation site present downstream of the exons encoding the Ig-like domain (Ashar-Patel et al., 2017). It gives rise to a shorter isoform that is soluble and not anchored to the membrane (Figure 2 E ). This isoform has known phenotypical effects, being the most abundant in placentas with growth deficiency (Young et al., 2010). This soluble isoform, known as sFLT1, has been proposed as both a treatment for different types of cancers (Zhu et al., 2011) and as a predictor for disease severity in Intrauterine Growth Restriction and Preeclampsia (Andrikos et al., 2022; Yamazaki et al., 2025). So, the gene is being transcribed and translated into two proteins: a pro-angiogenic and anti-angiogenic isoform, thereby controlling vascular growth in development and cancer in certain tissues. A similar distinction happens in other oncogenes listed in Table S1 (Day et al., 1999; Wasag et al., 2004; Albitar et al., 2010; Vander Ark et al., 2018; Mun et al., 2020; Mathur and Yee 2021) (Figure 2 E ).

Alternative isoforms also play decisive roles in shaping immune cell signaling. A classic example is *CD45* (now known as *PTPRC* - Protein Tyrosine Phosphatase Receptor Type C), a receptor-type protein tyrosine phosphatase expressed in all hematopoietic cells. Its extracellular domain is subject to extensive alternative splicing, generating isoforms with variable lengths (*CD45RA*, *RB*, *RC*, and *RO*) (Zilch et al., 1998; Oberdoerffer et al., 2008). These isoforms are not functionally redundant: they define thresholds of antigen receptor signaling in T cells, with naïve cells typically expressing high-molecular-weight variants (like *CD45RA*) (Henson et al., 2012), while memory and activated cells preferentially express shorter isoforms such as *CD45RO* (Tezuka et al., 1998; Hu and Wang 2017; Kwiecień et al., 2022). This isoform switch is essential for tuning T cell activation and differentiation. Members of the *TNF* (Tumor Necrosis Factor) receptor superfamily also produce isoform diversity through alternative splicing, with soluble variants functioning as antagonists of their membrane-anchored counterparts. For instance, soluble TNFR2 isoforms can sequester TNFα, attenuating inflammatory signaling, whereas membrane-bound forms propagate strong pro-inflammatory and apoptotic cues (Hauser et al., 2007; López-Urrutia et al., 2017; Wang *et al*., 2018), just like what happens to *VEGFR*. Caspase-9 (*CASP9*), a regulator of apoptosis and stress responses, produces isoforms with antagonistic functions. Its full-length isoform (caspase-9a) contains the catalytic domain required for apoptosome assembly and activation of downstream caspases. In contrast, an alternatively spliced variant lacking the catalytic domain (caspase-9b) acts as a dominant-negative inhibitor, binding to the apoptosome complex but preventing its activation (Shultz et al., 2010; Paronetto et al., 2016). This balance between caspase-9a and *caspase-9b* isoforms is crucial in determining whether a cell undergoes programmed death or evades apoptosis, especially on T-cells (Blake et al., 2022).

Transcription factors represent yet another layer of isoform-driven plasticity, where structural variation not only redefines DNA-binding specificity but can also confer dominant-negative properties or even RNA-binding capacity (Oksuz et al., 2023; Lambourne et al., 2025). While traditionally considered DNA-centric regulators, which bind to promoters and promote the transcription of that genomic region, an increasing number of TFs have been found to possess dual DNA- and RNA-binding properties, often mediated by distinct isoforms (Lambourne *et al*., 2025). This duality is frequently linked to the presence of intrinsically disordered regions (IDRs), which are highly enriched across TF families. IDRs provide flexible, low-specificity interfaces that can engage nucleic acids largely through electrostatic complementarity to the negatively charged phosphate backbone of both DNA and RNA (Már et al., 2023). In addition, stretches of positively charged residues such as lysine and arginine, even outside of IDRs, can stabilize interactions with nucleic acids and often define the strength and specificity of binding (Holehouse and Kragelund, 2024). Alternative splicing that alters the inclusion of these residues (whether within IDRs or structured domains) can, therefore, shift the balance between DNA and RNA recognition, creating isoforms that differentially regulate transcriptional and post-transcriptional programs. Such changes frequently extend beyond binding properties to influence subcellular localization: isoforms enriched for DNA-binding residues and nuclear localization signals tend to remain confined to the nucleus, where they regulate transcription, whereas variants retaining RNA-binding segments or lacking canonical localization motifs may accumulate in the cytoplasm, where they participate in RNA processing, stability, or translation control (Zeng and Hamada, 2020). In this way, splicing within IDR-rich or positively charged regions not only tune binding specificity but also partitions transcription factor isoforms between nuclear and cytoplasmic functions.

Another example, CTCF (CCCTC-Binding Factor) can bind to transcription activators and transcription repressors, but it is most well-known to act as an insulator protein, blocking or promoting the communication between enhancers and promoters. This gene is composed of 12 exons that code for multiple Zinc finger domains. An alternative isoform of *CTCF* (CTCF-s) where exons 3 and 4 are skipped can disrupt the CTCF binding to cohesin and to alter this chromatin loop, promoting a long-range interaction of *IFI6* gene enhancer and promoter, leading to cellular apoptosis (Li et al., 2019). This alternative use of Zinc finger binding domains is also present in other transcription factors. *WT1* (Wilms’ tumor Suppressor 1) isoforms with an exon inclusion between its zinc-finger domains show preferential interaction to unspliced mRNA in the polysomes when compared to its canonical, DNA binding activity (Bor et al., 2006). The deletion of this alternative *WT1* isoform impairs neuronal development in the olfactory system (Wagner et al., 2005), while its canonical isoform acts in the development of the urogenital system (Little et al., 1999). TP63 isoforms that lose its N-terminal transcription activation domain, can act as a dominant-negative, which turns them into an oncogenic promoting protein, blocking the canonical protein to bind to p53 (Chen et al., 2025 a ). Other examples such as FOXP1 (Forkhead Box P1) (van Keimpema et al., 2017) and STAT3 (Signal Transducer and Activator of Transcription 3) (Shamir et al., 2023) are present in Table S1.

In addition to transcription factors, RBPs themselves are frequently regulated through alternative splicing, creating isoforms with distinct, sometimes opposing, effects on RNA processing. A well-studied case is *PTBP1* and *PTBP2* (Polypyrimidine Tract Binding Protein 1/2), where isoform switching during neuronal differentiation alters splicing programs and enables the transition from proliferative to differentiated states (Vuong et al., 2016). Similarly, *hnRNPD* (Heterogeneous Nuclear Ribonucleoprotein D, previously known as *AUF1*) produces multiple isoforms with differential RNA stability functions, where some variants promote mRNA decay while others enhance transcript stability, shaping inflammatory and stress responses (Wilson et al., 1999; Banihashemi et al., 2006; Nechama et al., 2008). Another example is SRSF1(Serine and Arginine Rich Splicing Factor 1), a splicing factor whose alternatively spliced isoforms can act either as canonical splicing enhancers or as dominant-negative regulators that interfere with spliceosome assembly (Comiskey et al., 2015; Ivanova et al., 2023; Li et al., 2023).

These cases highlight that isoform diversification extends into the core machinery of RNA metabolism itself, establishing auto-reinforcing regulatory loops in which splicing factors are subject to the process they control. Alternative splicing of core splicing factors can, in turn, modulate the splicing of other genes, generating both coding isoforms through canonical and non-canonical pathways.

## Coding versus non-coding

Protein coding genes are also able to be transcribed into non-coding isoforms by alternative splicing and alternative start and termination points (Figure 2 A ). These alternative isoforms of coding genes collectively form a large and heterogeneous reservoir of transcriptional diversity that are not limited by the requirement to maintain an open reading frame (Deveson et al., 2018). While the coding isoforms diversify the proteome by producing structurally and functionally distinct proteins, the noncoding isoforms increase the functional RNA pool that regulates gene expression at multiple levels, with possible roles in transcriptional (Mohammad et al., 2010) and translational (Mahmoudi et al., 2009) regulation, splicing control (Tripathi et al., 2010), RNA stability (Peters et al., 2003), subcellular localization (Dumbović et al., 2021) and interactions with RNA-binding proteins (Tripathi *et al*., 2010), forming a complex and dynamic regulatory RNA network. In this way, noncoding isoforms represent an additional layer of transcriptome complexity, shaping cellular homeostasis and whose dysregulation has been implicated in numerous diseases that account for some of the highest mortality worldwide, including cancer, neurodegenerative disorders, and viral infections (Nemeth et al., 2024). 

The most striking condition known to disrupt cellular transcription and splicing and to promote the expression of multiple non-coding isoforms is viral infection. This occurs because many different viruses rely on interfering directly with the host spliceosome to express their complete transcriptional repertoire. Upon viral infection the cellular machinery can be extensively reprogrammed to reduce or redirect host splicing (Dias et al., 2024), generate proviral isoforms (Ku et al., 2011), antagonize interferon-stimulated genes (Miorin et al., 2020), or modulate apoptosis (Liu et al., 2015) and stress responses (Duarte et al., 2019). Viruses can influence host alternative splicing through three main mechanisms: (1) inducing global changes in splicing patterns during infection, (2) directly interacting with spliceosomal components, and (3) directly targeting splicing regulatory factors. Below, we present illustrative examples from both RNA and DNA viruses. Notably, RNA viruses display greater genome plasticity, and the alternative splicing changes reported during their infections are more diverse.

### Global impact on host alternative splicing

Upon viral infection, the host cell activates thousands of interferon-stimulated genes to combat the pathogen. In turn, viruses can reprogram the host’s alternative splicing, where global changes in hundreds of splicing events can modulate the production of pro- and antiviral isoforms, ultimately shaping infection outcomes. Human cytomegalovirus (HCMV) infection induces the expression of the host mRNA translation regulator CPEB1, leading to a global shortening of 3’ UTRs and lengthening of poly(A)-tails, which was shown to be necessary for viral replication (Batra et al., 2016). Epstein-Barr virus (EBV) infects resting B cells and induces alternative splicing changes that mimic cellular proliferation and activation, extending the lifespan of these short-lived cells to sustain long-term infection and further spread of the virus (Manet et al., 2021). During Rift Valley fever virus infection, the *RIOK3* (Right Open Reading Frame Kinase 3) mRNA undergoes alternative splicing, producing an isoform that inhibits the IFN-β pathway and weakens the antiviral response. This isoform, termed X2, uses an alternative splicing site in exon 8, resulting in the introduction of a premature termination codon (PTC) in exon 9. If translated, the protein lacks part of its putative kinase domain, but the transcript is likely degraded by means of NMD (White et al., 2022). Influenza A virus (FLUAV) induces global transcriptional defects at the 3′ ends of active host genes and RNA polymerase II run-through into extragenic regions (downstream-of-gene transcription), which leads to expression of aberrant RNAs that ultimately cause global transcriptional downregulation of physiological transcripts (Zhao et al., 2018 a ). FLUAV NS1 protein inhibits the pre‐mRNA splicing and blocks mRNA nucleocytoplasmic transport (Fortes et al., 1994). Lastly, Human Immunodeficiency Virus type 1 (HIV-1), along with other DNA-integrating viruses, can drive aberrant transcription of host genes adjacent to its integration site through a process in which viral RNA splices into host pre-mRNAs. This mechanism results in chimeric transcripts that disrupt normal host gene expression and can create novel isoforms (Liu et al., 2020).

### Direct interactions with spliceosome components

Many host spliceosomal proteins can be hijacked by viruses, where they are used either to alter host splicing patterns or to coordinate the splicing of viral transcripts. Herpes simplex virus type 1 (HSV-1) ICP27 protein interfaces with U2 snRNP and specifically re-distributes spliceosomal SNRPB protein (Phelan et al., 1993; Sandri-Goldin et al., 1995). Hepatitis C virus NS3 binds to the snRNP component Sm-D1 and re-localizes its protein to the nucleus (Iwai et al., 2003). SARS-CoV-2 Nsp16 protein binds to U1 and U2 snRNAs, hijacking the splice site recognition machinery and interfering with proper spliceosome assembly and function (Srivastava et al., 2020; Wang et al., 2022) leading to intron retention in genes involved in innate immune responses, including *RIG-I* (RNA Sensor RIG-I), *IRF7* (Interferon Regulatory Factor 7), and *ISG15* (Interferon-Stimulated Gene 15), thereby impairing antiviral signaling (Banerjee et al., 2020). Moreover, our group has shown that SARS-CoV-2 infection promotes unproductive splicing in key interferon signaling, interferon-stimulated (ISG), class I MHC, and splicing genes (e.g., *IRF7, HLA-B* (Major Histocompatibility Complex, Class I, B), *HNRNPH1* (Heterogeneous Nuclear Ribonucleoprotein H1) (Dias et al., 2024). Viral nucleic acids can also interact with snRNPs. For example, flavivirus sfRNAs bind the U2 snRNP component splicing factor 3b subunit 1 (SF3B1), which results in mis-splicing of host splicing factors *RBM5* (RNA Binding Motif Protein 5) (Tavanez et al., 2020), and *SRSF7* (Serine and Arginine Rich Splicing Factor 7) (Michalski et al., 2019), respectively.

### Interactions with splice regulatory factors

The trans-acting splice regulatory factors, SR (Serine/Arginine-rich) proteins and hnRNPs (Heterogeneous nuclear ribonucleoproteins), have many conserved domains which can be explored and co-opted during viral infections (Zahler et al., 1992). Members SRSF1 to SRSF3 (Serine And Arginine Rich Splicing Factor 1-3) are particularly affected, with infection often leading to a reduction in host splicing. For example, SRSF1 is upregulated during human papillomavirus type 16 (HPV16) infection (Klymenko et al., 2016) and interacts with the EB2 protein of EBV (Juillard et al., 2012). SRSF2 (Serine and Arginine Rich Splicing Factor 2) was shown to be re-localized by the HSV-1 ICP27 protein (Sandri-Goldin et al., 1995) and the FLUAV NS1 protein (Fortes et al., 1995). SRSF3, in turn, can be modulated either through re-localizing to virus-induced nuclear structures, as observed during EBV (Juillard *et al*., 2012) infection, or via cleavage of nucleoporins (Fitzgerald and Semler, 2011). HnRNPs are also predominantly affected during RNA virus infections, where they undergo degradation or are differentially localized to other intracellular components. HnRNP A1, for instance, is often redistributed to cytoplasm, as observed during infection with the enterovirus 71 (EV71), Sindbis virus (SINV) (Lin et al., 2009) or HIV-1 (Monette et al., 2009). HnRNP C can be co-opted by influenza B virus (FLUBV) to enhance viral replication (Patzina et al., 2017), whereas it exerts antiviral effects during reovirus infection (Dhillon et al., 2018). HnRNP D interacts with viral RNAs in EBV infections (Lee et al., 2012), and it is targeted for degradation by picornaviral 3CD protease, which shifts the viral RNA from translation to replication (Rozovics et al., 2012). Other hnRNPs, such as F, H1, K, M, and the U group, are similarly re-localized, bound, or degraded by viral proteins, affecting both antiviral responses and proviral processes (Lee *et al*., 2011; Pichlmair et al., 2012; Tsai et al., 2013; Thompson et al., 2018; Zhang et al., 2022).

### Neurodegenerative disorders and cancer

Most of the disease pathology studies outside viral infections do not assess the direct outcome of the differential transcription and splicing in a coding versus non-coding isoform manner. The nervous system exhibits the highest RNA isoform diversity amongst human tissues with multiple isoforms being exclusive to neuronal and glial cells (Mattick, 2023). Cancer is known to cause global hypomethylation in promoter CpG islands (Besselink et al., 2023) leading to disruption of transcription (Huang et al., 2020) and splicing regulation and the further production of various non-canonical transcripts and proteins, especially peptides coded from alternative ORFs (Zhang et al., 2025).

Michael J. Gandal’s group has advanced the understanding of splicing in neuropsychiatric disorders by moving beyond a gene-centric view to a transcript-level perspective, in the context of Autism Spectrum Disorder (ASD), Schizophrenia (SCZ), and Bipolar Disorder (BD) (Gandal *et al*., 2018; Bhattacharya et al., 2023). Splicing dysregulation is a pervasive feature of these diseases and it occurs through either disruption of the splicing machinery itself or altering production of specific coding and non-coding RNA isoforms. Changes at the isoform level showed larger effect sizes and greater disease specificity than changes in overall gene expression. Among the differentially spliced genes in ASD, SCZ or BD are the splicing factors (RNA binding proteins), including *MATR3* (Matrin 3), *QKI* (KH Domain-Containing RNA-Binding Protein QKI*)*, *RBM3* (RNA Binding Motif Protein 3), *SRRM2* (Serine/Arginine Repetitive Matrix 2), *U2AF1* (U2 Small Nuclear RNA Auxiliary Factor 1), and *SRSF11* (Serine And Arginine Rich Splicing Factor 11), suggesting impairment of the core splicing machinery (Gandal *et al*., 2018). Moreover, downstream targets of the neuronal splicing regulators *RBFOX1* (RNA Binding Fox-1 Homolog 1) and *FMRP* (Fragile X Mental Retardation Protein) - both strongly implicated in neurodevelopmental disorders - were also differentially spliced. This suggests a model where the detected genetic variation impairs splicing regulators, which subsequently dysregulate downstream genes (Li et al., 2016; Gandal *et al*., 2018). Differential transcript expression and local splicing is also documented in the psychiatric conditions (Gandal *et al*., 2018; Bhattacharya *et al*., 2023). The *GRIN1* (Glutamate Ionotropic Receptor NMDA Type Subunit 1) gene encodes the obligatory subunit of the N-methyl-D-aspartate receptor (NMDA) receptor, which is a type of glutamate receptor, the main excitatory neurotransmitter in the central nervous system. In ASD and SCZ, increased skipping of *GRIN1* exon 4 is observed, an event that directly alters the protein’s extracellular ligand-binding domain, likely resulting in an NMDA receptor isoform with an aberrant function (Gandal *et al*., 2018).

Furthermore, splicing dysregulation is a fundamental molecular feature of cancer, often acting as a primary driver of tumorigenesis rather than its consequence. This malfunction manifests through two primary mechanisms: mutations in the core spliceosome machinery itself and altered expression levels of regulatory splicing factors (Bradley and Anczuków, 2023). Recurrent mutations in core spliceosome genes act as driver events in cancer, in hematological malignancies (Yoshida et al., 2011). The most mutated gene is *SF3B1* (Splicing Factor 3b Subunit 1), a core part of the U2 snRNP complex (Dvinge et al., 2016). Mutations in hotspots of *SF3B1* alter its RNA-binding preference, causing the spliceosome to use cryptic, alternative branch points, which causes anomalous exon inclusion or skipping (Darman et al., 2015; Alsafadi et al., 2016). Functionally, this leads to the inclusion of a poison exon (an exon that contains an in-frame premature termination codon) in the tumor suppressor *BRD9* (Bromodomain Containing 9), resulting in its degradation and disruption of chromatin remodeling (Inoue et al., 2019). In solid tumors, splicing dysregulation commonly arises from the altered expression of splicing factors without mutation, shifting the homeostasis of isoform expression (Urbanski et al., 2018). The oncogenic SR protein SRSF1 is frequently overexpressed as a transcriptional target of the MYC (MYC Proto-Oncogene, BHLH Transcription Factor) oncogene (Das et al., 2012). Its overexpression promotes a pro-tumorigenic splicing program by generating isoforms that decrease apoptosis and increase proliferation (Anczuków *et al*., 2012). Another key example is SRSF3, which can be differentially expressed in a context dependent manner. It is a master regulator of the non-canonical metabolic switch in cancer cells, promoting the inclusion of exon 10 in the *PKM* gene to generate the *PKM2* isoform over the canonical *PKM1* (Wang et al., 2012). *PKM2* is a key driver of the Warburg effect (aerobic glycolysis) (Christofk et al., 2008). SRSF3 also regulates apoptosis through splicing of *HIPK2* (Kurokawa et al., 2014) and controls metabolic pathways, with its knockout in mice leading to hepatocellular carcinoma (Sen et al., 2015).

The production of specific, non-canonical protein isoforms that directly contribute to every stage of cancer progression is another consequence of splicing factor mutations and dysregulation. To sustain proliferative signaling, splicing of *RPS6KB1* (Ribosomal Protein S6 Kinase B1) generates a non-canonical isoform (S6K1-2) that activates mTORC1 signaling, promoting cell growth (Ben-Hur et al., 2013). Isoform-dependent functional opposition occurs in the *BCL2L1* (*BCL-X*) gene. Alternative splicing at the 5′ region of exon 2 generates two protein isoforms with antagonistic roles: *BCL-XL*, the long isoform, functions as an anti-apoptotic protein that stabilizes mitochondrial membranes and prevents cytochrome c release, whereas *BCL-XS*, the short isoform, lacks these domains and instead promotes apoptosis by facilitating cytochrome c release and caspase activation (Aird et al., 2019; reviewed in Chen and Li, 2021). This critical balance is shifted between the canonical anti-apoptotic *BCL-XL* isoform and the non-canonical pro-apoptotic *BCL-XS* isoform of *BCL2L1*, with cancer cells favoring survival (Boise et al., 1993). Also, cancer cells favor the full-length, active isoform of TERT (Telomerase Reverse Transcriptase) telomerase over inactive dominant-negative isoforms (Sayed et al., 2019). The circRNAs transcribed from protein-coding genes may also lack an open reading frame (ORF) yet still possess defined roles (reviewed in Kristensen et al., 2019). One such example is the exon-intron circRNA *circPAIP2* (Figure 2 D ), shown to enhance Pol II transcription of its parental gene by means of direct interaction with U1 snRNP (Li *et al*., 2015). *CircEIF3J* has been shown to do the same (Li *et al*., 2015), whereas others, such as *circUSP1* (Li *et al*., 2024b) and *circMMP9* (Li *et al*., 2024a) affect expression of other genes via different pathways.

Only very few protein-coding genes have been documented to generate both coding and non-coding isoforms with clearly established functions. In most cases, the literature instead contrasts coding versus non-coding transcripts, often assuming that the non-coding isoforms merely lack the regulatory activities attributed to their coding counterparts. Under methionine starvation, S-adenosylmethionine (SAM) depletion promotes removal of a retained intron in *MAT2A* (Methionine Adenosyltransferase 2A) pre-mRNA through the splicing of a 3’ UTR hairpin region. This intron retention switch ensures production of full-length *MAT2A* isoforms and restoration of SAM levels, linking splicing regulation to metabolic homeostasis (Pendleton et al., 2017). The *KCNMA1* (Potassium Calcium-Activated Channel Subfamily M Alpha 1) gene produces a retained-intron isoform that undergoes splicing under the presence of calcium in the cell. The 17a intron is timely spliced out allowing mRNA export to the cytoplasm and translation into the full-length calcium-activated big potassium channel (Bell et al., 2010). Additionally, a recent study using gradient fractionation followed by sequencing (GRAD-seq) in human and mouse iPSCs reveals that specific introns are retained in transcripts bound to stable RBP complexes, conferring them longer half-lives compared to other cellular RNAs (Pereira et al., 2025). This might represent nuclear “storage” of a pre-mRNA in preparation for a specific cellular signal or condition. In this case these RNAs are “poised” to be fully spliced and subsequently translated to protein, consistent with the two cases mentioned above. These few known examples show the retention of specific introns leading to temporal splicing and expression control, in which, upon stimulus, the intron is spliced out, exported and then the mRNA is translated. These alternative isoforms are also, since the loss of the ORF, a regulatory RNA and may be acting as such (Mattick et al., 2023). The most well-known class of regulatory RNAs are long non-coding RNAs and about half of them are transcribed by RNA Pol II. They also possess introns and exons and can also be regulated at the transcriptional level (Deveson et al., 2018).

## Long non-coding RNA isoforms

Long non-coding RNAs (lncRNAs) form a versatile class of non-coding RNAs (ncRNAs), heterogeneous both functionally and conservation-wise (Kapranov et al., 2007, reviewed in Mattick et al., 2023). Though there are noteworthy exceptions, rather than playing central roles in many cellular mechanisms, most seem to work as fine tuners of gene regulation in specific cell types or pathological and stress conditions (Adriaens and Marine, 2017). LncRNAs undergo the process termed universal alternative splicing, meaning that nearly all non-coding exons can be subject to alternative splicing in a modular fashion, in contrast to protein-coding genes’ exons which are constrained by the pressure of maintaining an open reading frame and therefore are more frequently prone to constitutive splicing. Thus, the maximum flexibility of lncRNAs in recombining their exons give way to great isoform diversity in comparison to protein-coding genes (Deveson et al., 2018).

Even though most lncRNAs are lowly expressed at basal levels and hardly conserved, a noteworthy exception is *NEAT1* (Nuclear-Enriched Abundant Transcript 1), and both its major isoforms have known and different roles inside the cell (Knutsen et al., 2022). These major isoforms are produced from the same promoter and generated by an alternative transcription termination point: *NEAT1_1* (or rather the very similar collection of isoforms that excludes NEAT1-202) which have around 3.7kb and *NEAT1_2* (NEAT1-202) which has around 23 kb. The long NEAT1 isoform, *NEAT1_2*, is fundamental for the assembly of paraspeckles, phase-separated ribonucleoprotein (RNP) bodies in the nucleoplasm (Figure 2 C ). Its knockout in mice, though viable, leads to impaired formation of corpus luteum and a concomitant reduced fertility (Nakagawa et al., 2014) and compromised mammary gland development (Standaert et al., 2014). Acting as a core structural scaffold, *NEAT1_2* recruits key paraspeckle proteins such as NONO, PSPC1, SFPQ, and DBHS (Naganuma et al., 2012). These membrane-less bodies have roles in nuclear events both in specific normal and pathological conditions, by means of binding and sequestering specific proteins and transcripts (Chen and Carmichael, 2009). While the short isoform (*NEAT1_1*) is pervasively more expressed, *NEAT1_2* expression is restricted to certain contexts; it has important roles in physiological conditions, such as the establishment of pregnancy and lactation (Nakagawa *et al*., 2014; Standaert *et al*., 2014).

*NEAT1* is differentially expressed in a wide range of tumors, and its upregulation is often correlated with poor prognosis (Adriaens et al., 2016). However, the role of NEAT1 in tumorigenesis remains ambiguous, as its isoforms may function either as tumor suppressors or oncogenes, with their effects varying across different solid tumor types (Knutsen et al., 2022). These different isoforms have also been reported to participate in the development of neurodegenerative diseases such as Parkinson’s disease. *NEAT1_2* can be upregulated during Parkinson’s progression and associated with protective effects against neurotoxicity (Simchovitz et al., 2019), but, incongruently, also reported to decrease and gradually exhibit a switch to the short isoform upon progression of the disease (Boros et al., 2025).

On the other hand, the short isoform, *NEAT1_1*, can be localized to structures independent from paraspeckles, named “microspeckles”, with undetermined functions (Li et al., 2017). Recent studies show that the short isoform also contributes to glucose metabolism in the cytoplasm. Upon high-glucose stimulation, it translocates from the nucleus and acts as a scaffold for assembling glycolytic complexes with enzymes PGK1, PGAM1, and ENO1. This promotes substrate channeling, thereby accelerating the pay-off phase of glycolysis. Through this mechanism, the short isoform supports the metabolic shift toward the Warburg effect, a hallmark of cancer cells (Park et al., 2021). 

Another example is *TUG1* (Taurine-Upregulated Gene 1), a 7.4 kb transcript with 3 exons. *Tug1* KO mice have notably been reported to be sterile, due to impaired spermatogenesis (Lewandowski et al., 2020). The retention of its introns 1 and 2 is known to cause its transcripts to be retained in the nucleus (Dumbović et al., 2021) (Figure 2 B ). While the nuclear *TUG1* isoforms regulate the expression of genes related to proliferation and apoptosis (reviewed in Zhou et al., 2019), its cytoplasmic counterpart may encode for a protein called TUG1-BOAT, by means of a conserved short open reading frame (sORF), that affects mitochondrial membrane potential when overexpressed (Lewandowski *et al*., 2020).

*LINC-PINT* (Long Intergenic Non-Protein-Coding RNA p53-induced Transcript) was first identified as a nuclear lincRNA regulated by p53 and has reported roles in promoting survival and acting as a tumor suppressor (Marín-Béjar et al., 2013). Its canonical isoform (LINC-PINT-215) is composed of 7 exons, but *LINC-PINT* also has a circular isoform, derived from its second exon, which can be translated via an Internal Ribosome Entry Site (IRES). This isoform is originated through backsplicing of exon 2, named *circPINTexon2*. This circular form harbors an IRES and encodes the peptide LINC87aa (Zhang et al., 2018). The expression of isoforms associated with *circPINTexon2* has been detected in multiple cell lines treated with Metformin, a drug with anti-carcinogenic properties (Conceição et al., 2022), and the presence of this peptide is able to control proliferation and exert tumor-suppressive effects when overexpressed in glioblastoma cells (Zhang *et al*., 2018).

Beyond the previously mentioned lncRNAs, many *GAS5* (Growth Arrest-Specific 5) isoforms have a peculiar feature when it comes to their structure. *GAS5* discovery dates back to 1988, when it was uncovered as a gene expressed in the quiescent phase of the cell cycle, G0 (Schneider et al., 1988). Since then, a one of its mechanisms of action has emerged: its exon 12 forms a hairpin structure capable of interacting with the DNA-binding domain of the glucocorticoid receptor (GR), mimicking target DNA elements canonically recognized by this receptor, the glucocorticoid response elements (GRE), therefore acting as a decoy riborepressor, resulting in competition for these receptors (Kino et al., 2010; Parsonnet *et al*., 2019). This mechanism is particularly relevant in both physiological and pathological contexts, given the role of glucocorticoid hormones in regulating immune function, metabolism, and stress response (Kino *et al*., 2010).

Extracellular vesicles can package not only proteins and full-length mRNAs isoforms, but also long non-coding transcripts (as well as short ncRNAs, most notably miRNA) (Figure 2 F ). These extend lncRNAs range of functions from intracellular fine tuners to that of far-reaching and systemic regulators (Kumar et al., 2024). An example is *linc001125*, an intergenic long non-coding found enriched in serum exosomes derived from non-small cell lung cancer patients, suggested as a biomarker for both its diagnosis and prognosis (Xian et al., 2021). Another mechanism for how lncRNAs can play a role in this sort of delivery is in the genesis of the transcripts that are packaged. One such instance is that of *circ_0076611*, which is formed from the backsplicing of *VEGFA* exon 7, an event dependent on lncRNA *MALAT1*. This circRNA seems to functionally promote a proliferative state in the tumour microenvironment and is detectable in exosomes in the serum of triple-negative breast cancer patients (Turco et al., 2022). Many others have been suggested as diagnostic and prognostic markers in cancer and are listed on Table S1 (Ruan et al., 2018; Zhao et al., 2018 b ; Yuan et al., 2019; Na-Er et al., 2021; Li et al., 2025; Zhao *et al*., 2025). Although most non-coding RNAs are typically not studied in their isoform-specific functional specialization, this section showcases the diversity of long non-coding RNA isoforms. They are often underestimated and challenging to analyze, but hold explanatory power for a broad landscape of phenotypic manifestations.

## Conclusion and upcoming

In recent years, accumulating evidence has shown that splicing is not limited to a co-transcriptional process but can also occur extensively post-transcriptionally. A recent review by Choquet, Patop and Churchman (Choquet et al., 2025) establishes post-transcriptional splicing as an underappreciated layer of gene regulation. Far from being a rare exception, delayed splicing of polyadenylated, chromatin-associated transcripts is now recognized as a widespread phenomenon, affecting more than one-third of mammalian introns. This temporal control allows transcripts to be maintained in a partially processed state, with their fate (productive splicing, nuclear retention, or targeted degradation) tuned to developmental stage, stress response, or disease context. Mechanisms such as detained introns, terminal intron processing, and nuclear speckle-associated regulation provide cells with precise means to synchronize protein output with physiological needs. The GRAD-seq work further supports this view, revealing that specific introns are retained in transcripts bound to stable RBP complexes, conferring longer half-lives to them when compared to other cellular RNAs. These findings suggest that retained-intron isoforms of coding genes may function not only as a transcriptional backlog but also as regulatory RNAs, in their own right (Pereira et al., 2025). The capacity to remove a single intron in response to defined cellular signals underscores the possibility of a fine-tuned alternative splicing code.

Beyond protein-coding and lncRNA isoforms, accumulating evidence highlights the importance of iso-miRs and other noncanonical RNA isoforms as key players in health and disease. Iso-miRs, sequence variants of canonical microRNAs, can alter target specificity, abundance, and regulatory networks, and have been implicated in cancer, immune dysfunction, and neurological disorders (Scheper et al., 2022; Wagner et al., 2024). Their stability in body fluids makes them particularly attractive for use in liquid biopsies as diagnostic or prognostic biomarkers. At the therapeutic level, several approaches are converging on isoform-specific modulation: antisense oligonucleotides (ASOs) to redirect splicing or silence pathogenic isoforms (El Marabti and Abdel-Wahab, 2021); SINE-UP RNAs to selectively enhance translation (Takahashi et al., 2018); and Perturb-seq-based strategies to systematically dissect isoform-specific functions at single-cell resolution (Dixit et al., 2016). Together, these tools open the possibility of tailoring interventions to the isoform level, providing a possible resource for precision medicine. By positioning isoforms as both biomarkers and therapeutic targets, the field is entering a stage where isoform biology may, in the future, directly inform prognosis, patient stratification, and the design of next-generation treatments. Ultimately, the study of isoforms reframes our view of the genome: genes do not encode single functions but dynamic repertoires of possibilities, regulated in time, space, and context. Harnessing this diversity represents a paradigm shift toward RNA isoform-centered molecular biology.

## Supplementary material

The following online material is available for this article:

Table S1 - List of all the isoforms mentioned on text, their references and further details

## Data Availability

No new data was generated in this project.
